# Systemic Inflammation Index and Tumor Glycolytic Heterogeneity Help Risk Stratify Patients with Advanced Epidermal Growth Factor Receptor-Mutated Lung Adenocarcinoma Treated with Tyrosine Kinase Inhibitor Therapy

**DOI:** 10.3390/cancers14020309

**Published:** 2022-01-08

**Authors:** Kun-Han Lue, Chun-Hou Huang, Tsung-Cheng Hsieh, Shu-Hsin Liu, Yi-Feng Wu, Yu-Hung Chen

**Affiliations:** 1 Department of Medical Imaging and Radiological Sciences, Tzu Chi University of Science and Technology, Hualien 970302, Taiwan; khlue@ems.tcust.edu.tw (K.-H.L.); shuhsin0816@tzuchi.com.tw (S.-H.L.); 2 Department of Nursing, Tzu Chi University, Hualien 970374, Taiwan; hou2017@gms.tcu.edu.tw; 3 Institute of Medical Sciences, Tzu Chi University, Hualien 970374, Taiwan; tchsieh@gms.tcu.edu.tw; 4 Department of Nuclear Medicine, Hualien Tzu Chi Hospital, Buddhist Tzu Chi Medical Foundation, Hualien 970473, Taiwan; 5 School of Medicine, College of Medicine, Tzu Chi University, Hualien 970374, Taiwan; 6 Department of Hematology and Oncology, Hualien Tzu Chi Hospital, Buddhist Tzu Chi Medical Foundation, Hualien 970473, Taiwan

**Keywords:** tyrosine kinase inhibitor (TKI), systemic inflammation index (SII), tumor heterogeneity, prognostic biomarker, epidermal growth factor receptor (*EGFR*), lung adenocarcinoma

## Abstract

**Simple Summary:**

Patients with advanced epidermal growth factor receptor (*EGFR*)-mutated lung adenocarcinoma have been known to respond to first-line tyrosine kinase inhibitor (TKI) treatment. However, a subgroup of patients are non-responsive to the treatment, with poor survival outcomes, and those who are initially responsive may still experience resistance. A reliable prognostic tool may provide a valuable direction for tailoring individual treatment strategies in this clinical setting. With this aim, the present study explores the prognostic power of the combination of the systemic inflammation index (portrayed by hematological markers) and tumor glycolytic heterogeneity (characterized by ^18^F-fluorodeoxyglucose positron emission tomography images). The model integrating these two biomarkers could be used to improve risk stratification, and the subsequent personalized management strategy in patients with advanced *EGFR*-mutated lung adenocarcinoma.

**Abstract:**

Tyrosine kinase inhibitors (TKIs) are the first-line treatment for patients with advanced epidermal growth factor receptor (*EGFR*)-mutated lung adenocarcinoma. Over half of patients failed to achieve prolonged survival benefits from TKI therapy. Awareness of a reliable prognostic tool may provide a valuable direction for tailoring individual treatments. We explored the prognostic power of the combination of systemic inflammation markers and tumor glycolytic heterogeneity to stratify patients in this clinical setting. One hundred and five patients with advanced *EGFR*-mutated lung adenocarcinoma treated with TKIs were retrospectively analyzed. Hematological variables as inflammation-induced biomarkers were collected, including the neutrophil-to-lymphocyte ratio (NLR), lymphocyte-to-monocyte ratio (LMR), platelet-to-lymphocyte ratio (PLR), and systemic inflammation index (SII). First-order entropy, as a marker of heterogeneity within the primary lung tumor, was obtained by analyzing ^18^F-fluorodeoxyglucose positron emission tomography images. In a univariate Cox regression analysis, sex, smoking status, NLR, LMR, PLR, SII, and entropy were associated with progression-free survival (PFS) and overall survival (OS). After adjusting for confounders in the multivariate analysis, smoking status, SII, and entropy, remained independent prognostic factors for PFS and OS. Integrating SII and entropy with smoking status represented a valuable prognostic scoring tool for improving the risk stratification of patients. The integrative model achieved a Harrell’s C-index of 0.687 and 0.721 in predicting PFS and OS, respectively, outperforming the traditional TNM staging system (0.527 for PFS and 0.539 for OS, both *p* < 0.001). This risk-scoring model may be clinically helpful in tailoring treatment strategies for patients with advanced *EGFR*-mutated lung adenocarcinoma.

## 1. Introduction

Lung cancer is the leading cause of cancer-related death, causing an estimated 1.8 million deaths worldwide in 2020 [1]. It consists of different subtypes that can be classified histopathologically. Adenocarcinoma is the most frequent subtype of non-small cell lung cancer (NSCLC), and the majority of patients with lung adenocarcinoma have a poor prognosis [2,3]. Fortunately, the testing of epidermal growth factor receptor (*EGFR*) alterations serves as a targeted therapy recommendation to guide treatment selection in patients with lung adenocarcinoma [4]. Targeted tyrosine kinase inhibitors (TKIs) have been introduced as a first-line regimen for the treatment of advanced *EGFR*-mutated lung adenocarcinoma [5,6]. TKIs improve patient outcomes compared with standard chemotherapy. However, a subgroup of patients are non-responsive to TKI, and those who are initially responsive may still experience resistance within 12 months [7]. A personalized therapeutic strategy is urgently needed to improve the survival outcomes in patients with advanced *EGFR*-mutated lung adenocarcinoma.

Systemic inflammation is a key element of the survival outcome for numerous types of cancer [8,9]. It promotes tumor-promoting activities, including angiogenesis, mutagenesis, and immunosuppression [10]. Hematological parameters, such as the neutrophil-to-lymphocyte ratio (NLR) and systemic inflammation index (SII), have prognostic roles in various malignancies, including lung cancer, as potential cancer inflammation-induced biomarkers [11,12,13,14,15,16]. These peripheral blood parameters are of notable interest because of their easy and ready accessibility in clinical practice [17]. Meanwhile, ^18^F-fluorodeoxyglucose positron emission tomography (^18^F-FDG PET) imaging is the standard imaging modality for the staging, response assessment, and follow-up processes of lung cancer [18,19]. ^18^F-FDG PET reflects the glycolytic activity of tumor cells associated with malignant signaling pathways [20]. The literature has shown that several semiquantitative measurements of radioactivity concentration have been associated with patient prognosis in NSCLC [21]. Further, tumor heterogeneity is a crucial factor that correlates with tumor aggressiveness and metastasis in cancers [22]. Quantitative texture features derived from ^18^F-FDG PET images, such as first-order entropy, allow the assessment of glycolytic heterogeneity within the tumor [23,24]. Distinct from the clinical profile and cancer staging system, which pictures the disease to a greater extent, texture analysis portrays more associations with aspects of tumor behavior. An increasing number of recent studies have demonstrated that tumor entropy is a robust predictor of survival in patients with lung cancer [25,26,27,28,29]. However, little is known about the comparison and potential combinations of hematologic inflammation biomarkers and tumor glycolytic heterogeneity in the current clinical setting.

Awareness of a reliable prognostic tool may provide a valuable direction for tailoring individual treatments for *EGFR*-mutated NSCLC, especially adenocarcinoma. Therefore, this study aimed to explore the prognostic power of the combination of systemic inflammation markers and tumor glycolytic heterogeneity for the risk stratification of patients with advanced *EGFR*-mutated lung adenocarcinoma treated with a first-line TKI.

## 2. Materials and Methods

### 2.1. Study Patients

Patients with a histopathological diagnosis of *EGFR*-mutated lung adenocarcinoma between March 2010 and December 2017 were retrospectively enrolled. The electronic charts were carefully reviewed for each patient. Data regarding patient demographics, smoking status, staging, mutation type, and survival outcomes were recorded. Patients with a documented history of smoking were classified as ever-smokers. Patients with no smoking history were classified as never-smokers. All patients underwent serial imaging studies at the initial stage of diagnosis, including thoracic to upper abdominal computed tomography (CT), ^18^F-FDG PET/CT, and brain magnetic resonance imaging (MRI). Staging was performed according to the seventh edition of the American Joint Committee on Cancer staging system [30]. Using an *EGFR* RGQ Kit (Qiagen, Hilden, Germany), *EGFR* mutational analysis was performed on formalin-fixed, paraffin-embedded tissues of histopathologically confirmed lung adenocarcinoma. All patients had stage IIIB or IV disease with an active *EGFR* mutation in exons 18, 19, 20, or 21. These patients received *EGFR*-targeting TKIs as first-line treatment, including gefitinib, erlotinib, or afatinib. The choice of TKI was based on the decision of the attending physician. Our study was conducted in accordance with the Declaration of Helsinki, and the protocol was approved by the institutional review board and ethics committee. Given the retrospective nature of the study, the need for informed consent was waived.

### 2.2. Treatment and Follow-Up

The initial TKI treatment was administered to patients with *EGFR*-mutated lung adenocarcinoma according to the National Comprehensive Cancer Network Clinical Practice Guidelines. The disease status of patients was evaluated following treatment at the outpatient clinic once every month. Chest CT with contrast enhancement was performed every 3 months. Biopsy of a suspicious lesion and MRI of the brain were performed if the disease symptoms of progression emerged. The results of the imaging studies and treatment strategies were discussed at a conference held by our thoracic oncology group. All patients were followed up until disease progression or death, and cases were counted as events. Progression-free survival (PFS) was defined as the time from the initiation of TKI treatment to the date of disease progression or death. Overall survival (OS) was defined as the time from diagnosis to the date of death. Patients who did not experience any event were censored at the last follow-up until December 2020.

### 2.3. Systemic Inflammation Biomarkers

Routine blood tests were performed on all patients prior to the initiation of TKI treatment. Complete blood counts included white blood cell count, platelet count, and hemoglobin levels from the peripheral blood samples in the laboratory examination. Absolute neutrophil, lymphocyte, and monocyte counts were also assessed. Hematological inflammation biomarkers were defined as follows: NLR, neutrophil count/lymphocyte count; PLR, platelet count/lymphocyte count; LMR, lymphocyte count/monocyte count; and SII, platelet count × neutrophil count/lymphocyte count [31].

### 2.4. Tumor Glycolytic Heterogeneity

^18^F-FDG PET/CT scans were performed for all patients before TKI treatment using a GE Discovery ST scanner (GE Healthcare, Milwaukee, WI, USA). The scan procedure followed the guidelines of the European Association of Nuclear Medicine for tumor imaging [32]. An expert nuclear medicine physician interpreted the ^18^F-FDG PET images and identified the primary tumor of the lung adenocarcinoma. All images were analyzed by the same reviewer using the PMOD image processing software version 4.2 (PMOD Technologies Ltd., Zurich, Switzerland) to avoid interobserver variability. A standardized uptake value (SUV) threshold above 2.5 was adopted for target contouring [33]. The SUV-based volumes of interest were used to estimate entropy as a feature of tumor glycolytic heterogeneity. The entropy was computed based on a first-order histogram with 64 quantization bins [34]. The Pyradiomics open-source software package version 3.0.1 was used to calculate the entropy values complying with the texture feature definitions described by the Imaging Biomarker Standardization Initiative [35].

### 2.5. Statistical Analysis

Clinical variables, hematological inflammation biomarkers, and ^18^F-FDG-derived entropy were assessed for their correlation with PFS and OS. Cox proportional hazard regression models were used to identify the prognostic factors of PFS and OS. Statistically significant variables in the univariate Cox analysis were included in the stepwise multivariate Cox regression model to identify the independent factors of survival. The results of the survival analysis were expressed as hazard ratios and 95% confidence intervals (CIs). The continuous variables of hematologic markers and SUV entropy were not dichotomized while performing regression analyses. After identifying the independent prognostic factors, the X-tile bioinformatics software (Yale University, New Haven, CT, USA) was used to define the optimal cut-off using the maximum chi-squared value and minimum *p*-value for continuous biomarkers [36]. The survival curve was plotted using the Kaplan–Meier method, and the survival difference between subgroups was estimated using a log-rank test. The prognostic scoring model for PFS and OS was constructed based on the independent risk factors. The prognostic performance of the models was evaluated using Harrell’s C-index [37]. The model was validated using a bootstrapping method for internal validation. The validation process was performed using 1000 bootstrap samples. Bootstrapping validation results were expressed as bias with standard error (SE) and significance (*p*-value). All statistical tests were two-sided, and the significance level was set at a *p*-value of < 0.05. Statistical analyses were conducted using MedCalc 20.014 (MedCalc Software, Ostend, Belgium) and R 4.0.3 (R Foundation, Vienna, Austria).

## 3. Results

### 3.1. Patient Characteristics

A total of 105 patients met the criteria for enrolment in the study. Adenocarcinoma was histopathologically confirmed in all patients. Of these, 16 patients were initially diagnosed with stage IIIB disease and 89 with stage IV disease. Regarding the type of *EGFR* mutation, 50 patients had a deletion in exon 19, 50 had exon 21 L858R mutation, and 5 had uncommon mutations, including 1 with S768I in exon 20, 2 with L861Q in exon 21, and 2 with G719X mutations. The clinical characteristics of the patients are summarized in Table 1. The median follow-up period was 22.5 months (interquartile range, 14.9–32.3 months). At the time of the last known follow-up, 84 patients (80.0%) experienced disease progression at a median of 14.4 months after TKI treatment, and 65 patients (61.9%) died of the disease at a median of 28.3 months. The 3-year PFS rate was 13.3%, and the 3-year OS rate was 39.9% in the entire study population.

### 3.2. Prognostic Factors for Survival Endpoints

The results of the univariate and multivariate Cox regression analyses for the clinical variables, hematologic markers, and tumor entropy are presented in Table 2 and Table 3, respectively. In the univariate analysis, sex, smoking status, NLR, LMR, PLR, SII, and entropy were associated with PFS and OS. These variables were entered into a multivariate Cox regression model. After adjusting for confounders in the multivariate analysis, smoking status, SII, and entropy remained prognostic factors for PFS and OS.

**Figure 1:**
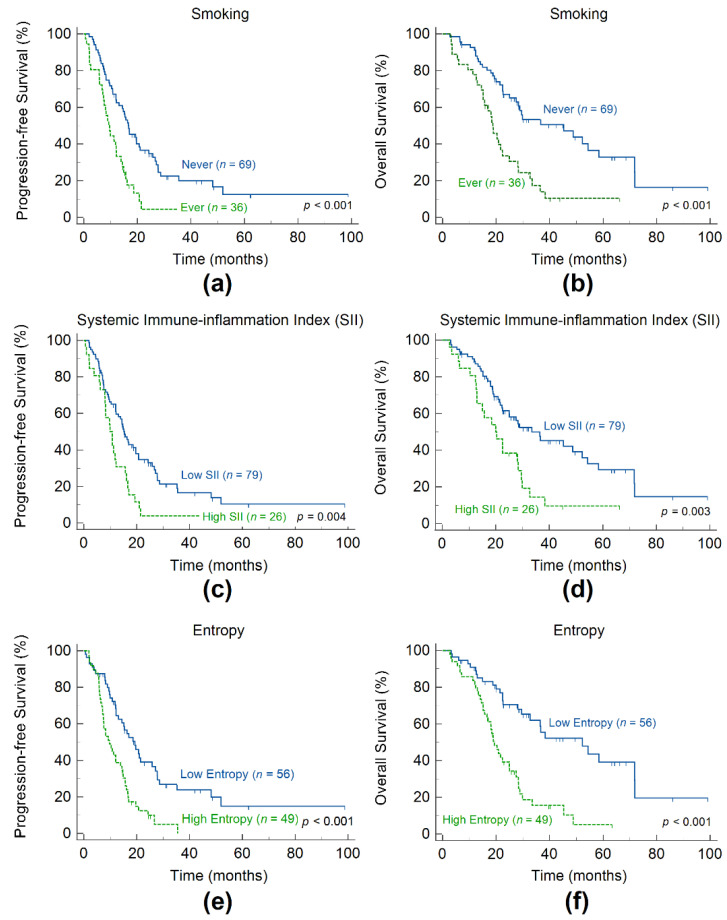
Kaplan–Meier estimates of progression-free survival and overall survival according to the patient smoking status (**a**,**b**), systemic inflammation index (**c**,**d**), and tumor entropy (**e**,**f**).

Figure 1 depicts the Kaplan–Meier survival plots based on the independent prognostic factors. The cut-off value of SII was 1296, and entropy was 5.35, which was used to stratify patients into those with good or poor survival outcomes. The 3-year estimate of PFS was 4.41% in the ever-smoking group compared with 20.0% in the never-smoking group. Patients who smoked had a 3-year OS of 17.5%, and those who did not smoke had a 3-year OS of 50.6%. Moreover, patients with a high SII had a 3-year PFS of 3.85%, whereas patients with a low SII had a 3-year PFS of 16.6%. Patients with a high SII had a 3-year OS of 14.4%, whereas patients with a low SII had a 3-year OS of 50.0%. Patients with high entropy had a greater risk of disease progression and a lower overall survival rate than those with low entropy. Patients with high entropy had a 3-year PFS of 0%, compared with 23.9% of patients with low entropy. Patients with high entropy had a 3-year OS of 15.6%, whereas patients with low entropy had a 3-year OS of 62.0%.

### 3.3. Development and Validation of Prognostic Scoring Model

A prognostic model was constructed based on the independent risk factors presented in the multivariate Cox regression analysis. The risk factors included smoking status from the clinical variables, high SII from the hematological markers, and high entropy from the texture feature. By combining the three factors, a prognostic score was calculated for each patient using the regression coefficient-based (Schneeweiss) scoring system [38]. Table 4 shows the weighted scores of each prognostic factor defined by the β-coefficients of the Cox regression models. The scoring system ranged from 0 to 7 points for PFS and 0 to 9 points for OS.

**Figure 2:**
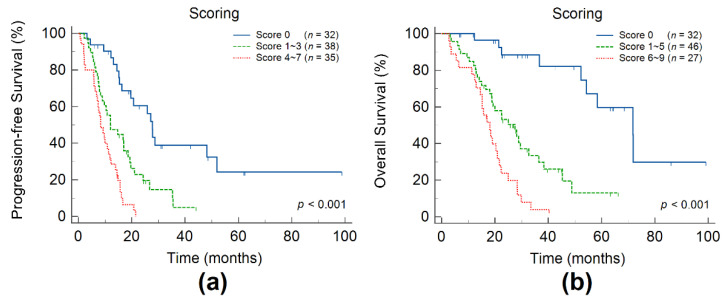
Kaplan–Meier estimates of progression-free survival (**a**) and overall survival (**b**) according to the prognostic scoring model.

The patients were divided into three risk groups based on their prognostic scores. The scores for the low-, intermediate-, and high-risk groups were 0, 1–3, and 4–7 for PFS and 0, 1–5, and 6–9 for OS, respectively. PFS and OS were subjected to Kaplan–Meier analysis to evaluate the ability of the risk-scoring system (Figure 2). Survival curves showed significant differences in PFS and OS among the three risk groups. The 3-year PFS rates of patients in the low-, intermediate-, and high-risk groups were 38.9%, 4.90%, and 0% (*p* < 0.001), respectively, and the 3-year OS rates were 88.4%, 33.5%, and 3.97% (*p* < 0.001), respectively.

The scoring model achieved a C-index of 0.687 (95% CI: 0.637–0.738) in predicting PFS, which was significantly higher than that of smoking status (C-index: 0.597, 95% CI: 0.547–0.646, *p* = 0.013), SII (C-index: 0.595, 95% CI: 0.528–0.661, *p* = 0.030), and entropy (C-index: 0.592, 95% CI: 0.534–0.650, *p* = 0.019) factors. For OS, the integrated model had a C-index of 0.721 (95% CI: 0.666–0.776), which was significantly higher than that of smoking status (C-index: 0.617, 95% CI: 0.557–0.676, *p* = 0.012), SII (C-index: 0.619, 95% CI: 0.543–0.695, *p* = 0.033), and entropy (C-index: 0.620, 95% CI: 0.554–0.686, *p* = 0.021). The prognostic scoring model outperformed the traditional TNM staging system in predicting PFS (C-index, 0.527; 95% CI: 0.488–0.567, *p* < 0.001) and OS (C-index: 0.539, 95% CI: 0.500–0.678, *p* < 0.001), respectively.

The prognostic model based on risk scoring was further validated using a bootstrap validation method. The validation process was performed using 1000 bootstrap samples consisting of random samples gathered from the original sample with replacement. Table 5 presents the bootstrap validation results. The β-coefficient estimates of the three independent factors remained statistically significant in predicting both PFS and OS after validation. The β-coefficients and SE estimated from the bootstrap samples were remarkably comparable to those calculated from the original Cox regression models, suggesting excellent internal validation (Table 4 and Table 5).

## 4. Discussion

The present study investigated the combination of systemic inflammation and tumor heterogeneity biomarkers in predicting survival outcomes in patients with advanced *EGFR*-mutated lung adenocarcinoma treated with a first-line TKI. To the best of our knowledge, this is the first study to represent the simultaneous analysis of inflammatory biomarkers and tumor glycolytic heterogeneity as prognostic factors in this clinical setting. Our findings suggest that SII and entropy have independent prognostic values for both PFS and OS. It can be reasoned that cancer-induced inflammation and tumor glycolytic heterogeneity are closely associated with clinical outcomes. Furthermore, a prognostic scoring model was constructed to determine risk groups based on the integration of SII and entropy with smoking status. The integrated model outperformed the TNM system and allowed further stratification of survival outcomes into three risk groups of patients with advanced *EGFR*-mutated lung adenocarcinoma.

Many studies have attempted to identify reliable prognostic biomarkers to improve the management of patients with cancer [39]. Inflammation is considered a hallmark of malignancy. SII is a novel inflammation-related biomarker that comprehensively combines peripheral lymphocyte, neutrophil, and platelet counts. Lymphocytes are involved in the host defense mechanisms against cancer cells, whereas neutrophils and thrombocytes are reported to play critical roles in tumor invasion, proliferation, cancer cell survival, and metastasis. Hence, SII reflects the balance between host inflammatory and immune response conditions [40]. Recent evidence suggests that pretreatment SII may serve as a useful prognostic indicator in patients with NSCLC [12,13,41,42,43,44,45,46]. Our results are consistent with those of findings that showed SII is associated with the prognosis of clinical outcomes. Concerning tumor heterogeneity, ^18^F-FDG PET-derived texture features have been shown to be correlated with genetic heterogeneity and prognostic outcomes [47,48]. The present study used primary tumor SUV entropy as a surrogate marker for heterogeneity, specifying randomness and uncertainty in tumor glycolytic activity. Entropy has been reported to be the most stable and reproducible feature of radiomic signatures [49,50,51]. Therefore, we selected entropy as the tumor heterogeneity feature in this study. In addition, extracting entropy features reflects entire tumor heterogeneity, which may avoid the potential drawback of single-site biopsy. Regarding the clinical history of smoking status, the majority of reports have shown that never-smokers have a favorable prognosis for survival outcomes in lung cancer over that of ever-smokers [52,53,54,55,56]. These results add to a growing body of evidence implicating smoking as an independent risk factor for lung cancer. Taken together, the current study can be considered a validation study of previously published data.

**Figure 3:**
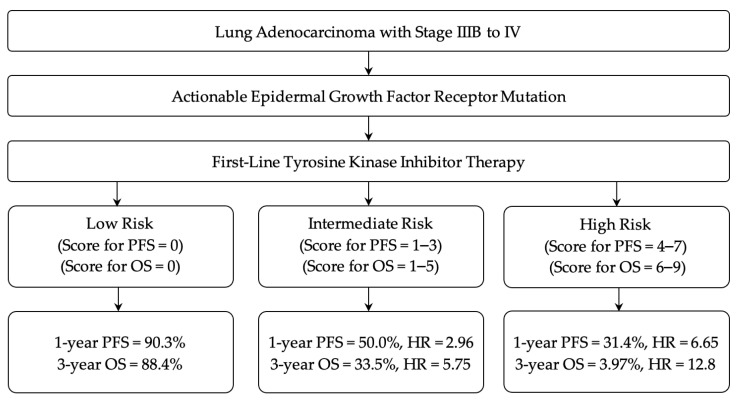
Flow chart illustrating the potential utility of the prognostic scoring system in the management of patients with epidermal growth factor receptor-mutated lung adenocarcinoma treated with tyrosine kinase inhibitor. PFS, progression-free survival, OS, overall survival, HR, hazard ratio.

The above findings lead to the development of a prognostic scoring model that allows clinicians to obtain more comprehensive information on therapeutic strategy management in advanced *EGFR*-mutated lung adenocarcinoma. These three biomarkers may play complementary roles in predicting the survival prognosis and management of patients. Integration of SII and entropy with smoking status paved the way to construct a risk-scoring system and improve survival stratification (Figure 3). Recently, therapeutic approaches combined with immune modulation have been designed, and show durable responses in patients with actionable *EGFR* mutations [57]. Our scoring model could be used to identify different risk groups of patients who are suitable for novel treatment strategies. In contrast, patients with an excellent response to first-line TKI therapy may omit add-on therapies to facilitate personalized precision medicine. Moreover, the SII is an inexpensive and easily measurable laboratory variable. ^18^F-FDG PET is widely used; hence, the SUV entropy feature is readily available. These advantages may further enhance the applicability of our prognostic scoring system in clinical practice.

Regarding statistical considerations, continuous variables (i.e., NLR, LMR, PLR, SII, and SUV entropy) were estimated without dichotomization in the univariate and multivariate Cox regressions. In this study, we aimed to explore whether a biomarker was singly prognostic. Evaluating the biomarker without a pre-selected cut-off point is the preferred approach before regression analysis. Such a method has the advantage of retaining valuable information in the data and avoiding an inflated effect on the regression model [58]. After multivariable Cox regression analyses, independent prognostic factors were combined to form a risk-scoring system. The choice of a cut-off for the continuous prognostic index was introduced at this time point. The cut-off value was used to categorize patients into subgroups with distinct prognoses. The composite risk score was computed for individual patients by adding a weighted score based on the estimated Cox regression coefficients [38]. The risk score represents a new variable that can be used for prognostication. Three risk groups were defined according to the range of prognostic risk scores using our model. The above statistical processing may allow us to compare the regression results from other studies directly, and could increase the usefulness of the proposed scoring system in clinical settings.

With respect to sensitizing mutations in *EGFR*, evidence has suggested that T790M mutations are found in approximately 50% of *EGFR*-mutated patients who acquired resistance to first-line TKIs [59]. Our study lacked secondary testing data for the *EGFR* T790M mutation. A third-generation TKI, osimertinib, was developed to overcome T790M mutation-induced resistance [60]. Future prospective studies should incorporate the T790M mutation type into our scoring model. In addition, a recent study revealed that the *EGFR* variant allele frequency (*EGFR*-aVAF) of tumor tissue can predict the benefit of TKI treatment in advanced lung adenocarcinoma [61]. Lower actionable *EGFR*-aVAF was correlated with unfavorable survival outcomes. Since a lower *EGFR*-aVAF indicates that fewer cells harbor this driver mutation, the tumor may be more heterogeneously mixed with other mutations that drive carcinogenesis. ^18^F-FDG PET-derived entropy was used as a surrogate for tumor heterogeneity in our study. Thus, entropy and *EGFR*-aVAF may have similar predictive values in our patient cohort. Further work is needed to explore the association between biological meaning and its effect on the outcome.

We acknowledge that our study is exploratory and has some limitations. First, the cut-off point was selected to categorize the patients in our scoring system. The cut-off points of the reported biomarkers are often inconsistent [13,15]. This may hinder the adoption of potential prognostic factors in clinical practice. Further large cohort trials need to be conducted to identify an optimal cut-off value of continuous variables of biomarkers (i.e., SII and entropy). Second, a subset analysis of smokers could not be carried out in the present study due to a lack of detailed information, such as the numbers of current smokers, former smokers, and smoking pack-years. Third, because of the retrospective nature of our study, inherent selection bias could not be neglected. Additionally, our analysis was based on a small number of patients from a single center. Data have shown that patients with *EGFR* exon 19 deletion have longer PFS and OS rates after *EGFR*-TKI therapy than those with the L858R mutation [62,63]. In our study, the absence of PFS and OS benefits may have resulted from TKI variability and an insufficient number of patients. The differences between the subgroups of TKIs according to mutation type should be analyzed in a large cohort study. Fourth, tumor entropy is a visually imperceptible feature. Why the entropy feature is associated with specific pathways remains unexplored, and its underlying biological characteristics need further elucidation [64]. Finally, although an internal validation was performed in our study, the generalizability of our findings requires prospective validation in a large external cohort.

## 5. Conclusions

Systemic inflammation index and tumor glycolytic heterogeneity have independent prognostic values for survival outcomes in patients with advanced *EGFR*-mutated lung adenocarcinoma treated with TKIs. Integrating these two biomarkers with smoking status represents a valuable prognostic scoring tool for improving the risk stratification of patients. The risk-scoring model may be clinically helpful in tailoring treatment strategies in patients with advanced *EGFR*-mutated lung adenocarcinoma.

## Figures and Tables

**Table 1:** Baseline patient characteristics (*n* = 105).

Characteristic	Value
Age, median (IQR), years	70 (16)
Sex, *n* (%)	
Male	48 (45.7)
Female	57 (54.3)
Cigarette smoking status, *n* (%)	
Ever-smoker	36 (34.3)
Never-smoker	69 (65.7)
Mutation type of *EGFR*, *n* (%)	
Deletion 19	50 (47.6)
L858R	50 (47.6)
Others	5 (4.8)
Overall stage, *n* (%)	
Stage IIIB	16 (15.2)
Stage IV	89 (84.8)
Pleural effusion, *n* (%)	36 (34.3)
Brain metastasis, *n* (%)	23 (21.9)
First line TKI, *n* (%)	
Gefitinib	48 (45.7)
Erlotinib	30 (28.6)
Afatinib	27 (25.7)
Time from ^18^F-FDG PET to TKI treatment, median (IQR), days	12 (25)

*EGFR*, epidermal growth factor receptor; TKI, tyrosine kinase inhibitor; IQR, interquartile range.

**Table 2:** Univariate and multivariate Cox regression for prognostic factors of progression-free survival.

Variable	Univariate Analysis		Multivariate Analysis	
	HR (95% CI)	*p*-Value	HR (95% CI)	*p*-Value
Age > 70 years (median)	1.327 (0.859–2.050)	0.201		
Sex (male vs. female)	1.739 (1.117–2.707)	0.014 *	1.239 (0.716–2.142)	0.443
Smoking (ever vs. never)	2.442 (1.540–3.871)	<0.001 *	2.235 (1.405–3.556)	<0.001 *
Mutation (Del 19 vs. others)	1.093 (0.710–1.682)	0.684		
Overall stage (IIIB vs. IV)	1.813 (0.921–3.567)	0.084		
Pleural effusion (yes vs. no)	1.213 (0.775–1.899)	0.397		
Brain metastasis (yes vs. no)	1.060 (0.640–1.755)	0.820		
Hematologic makers ^#^				
NLR	1.050 (1.010–1.091)	0.013 *	0.978 (0.891–1.073)	0.645
LMR	0.883 (0.803–0.972)	0.011 *	0.977 (0.883–1.083)	0.664
PLR	1.002 (1.000–1.003)	0.008 *	1.000 (0.997–1.003)	0.799
SII ^%^	1.166 (1.063–1.279)	0.001 *	1.166 (1.058–1.286)	0.002 *
PET SUV entropy ^#^	2.270 (1.308–3.939)	0.003 *	2.093 (1.188–3.687)	0.011 *

HR, hazard ratio; CI, confidence interval; NLR, neutrophil-to-lymphocyte ratio; LMR, lymphocyte-to-monocyte ratio; PLR, platelet-to-lymphocyte ratio; SII, systemic inflammation index; PET, positron emission tomography; SUV, standardized uptake value; *, statistically significant; ^#^, continuous variable; ^%^, normalized to 1000 counts.

**Table 3:** Univariate and multivariate Cox regression for prognostic factors of overall survival.

Variable	Univariate Analysis		Multivariate Analysis	
	HR (95% CI)	*p*-Value	HR (95% CI)	*p*-Value
Age > 70 years (median)	1.617 (0.984–2.656)	0.057		
Sex (male vs. female)	2.128 (1.275–3.552)	0.003 *	1.682 (0.867–3.261)	0.123
Smoking (ever vs. never)	2.664 (1.607–4.415)	<0.001 *	2.259 (1.356–3.761)	0.001 *
Mutation (Del 19 vs. others)	1.533 (0.930–2.524)	0.093		
Overall stage (IIIB vs. IV)	2.191 (0.981–4.891)	0.055		
Pleural effusion (yes vs. no)	1.373 (0.835–2.259)	0.211		
Brain metastasis (yes vs. no)	0.896 (0.486–1.652)	0.726		
Hematologic makers ^#^				
NLR	1.055 (1.012–1.101)	0.012 *	0.931 (0.840–1.032)	0.172
LMR	0.815 (0.720–0.922)	0.001 *	0.920 (0.793–1.067)	0.269
PLR	1.002 (1.000–1.003)	0.003 *	1.001 (0.997–1.004)	0.747
SII ^%^	1.222 (1.102–1.355)	<0.001 *	1.215 (1.088–1.356)	<0.001 *
PET SUV entropy ^#^	3.380 (1.664–6.868)	<0.001 *	3.422 (1.589–7.370)	0.001 *

HR, hazard ratio; CI, confidence interval; NLR, neutrophil-to-lymphocyte ratio; LMR, lymphocyte-to-monocyte ratio; PLR, platelet-to-lymphocyte ratio; SII, systemic inflammation index; PET, positron emission tomography; SUV, standardized uptake value; *, statistically significant; ^#^, continuous variable; ^%^, normalized to 1000 counts.

**Table 4:** Multivariate Cox regression coefficients and prognostic scoring definition.

Variable	Progression-Free Survival			Overall Survival		
	β-Coefficient ± SE	*p*-Value	Score ^#^	β-Coefficient ± SE	*p*-Value	Score ^#^
Smoking (ever)	0.718 ± 0.239	0.003 *	2	0.692 ± 0.261	0.008 *	2
SII (>1296)	0.730 ± 0.248	0.003 *	2	0.907 ± 0.279	0.001 *	3
Entropy (>5.35)	0.895 ± 0.238	<0.001 *	3	1.248 ± 0.283	<0.001 *	4

SE, standard error; SII, systemic inflammation index; *, statistically significant; ^#^, weighing scheme based on Schneeweiss’ scoring system [38].

**Table 5:** Bootstrap validation of multivariate Cox regression models.

Variable	Progression-Free Survival		Overall Survival	
	β-coefficient ± SE	*p*-Value	β-coefficient ± SE	*p*-Value
Smoking (ever)	0.718 ± 0.236	0.002 *	0.692 ± 0.278	0.006 *
SII (>1296)	0.730 ± 0.260	0.001 *	0.907 ± 0.308	0.005 *
Entropy (>5.35)	0.895 ± 0.237	0.001 *	1.248 ± 0.300	0.001 *

SE, standard error; SII, systemic inflammation index; *, statistically significant.

## Data Availability

The data presented in this study are available on request from the corresponding author. The data are not publicly available due to the privacy and ethical restrictions.

## References

[1] Sung H., Ferlay J., Siegel R.L., Laversanne M., Soerjomataram I., Jemal A., Bray F. (2021). Global Cancer Statistics 2020: GLOBOCAN Estimates of Incidence and Mortality Worldwide for 36 Cancers in 185 Countries. CA Cancer J. Clin..

[2] Barta J.A., Powell C.A., Wisnivesky J.P. (2019). Global Epidemiology of Lung Cancer. Ann. Glob. Health.

[3] Tsim S., O’Dowd C.A., Milroy R., Davidson S. (2010). Staging of non-small cell lung cancer (NSCLC): A review. Respir. Med..

[4] Lindeman N.I., Cagle P.T., Aisner D.L., Arcila M.E., Beasley M.B., Bernicker E.H., Colasacco C., Dacic S., Hirsch F.R., Kerr K. (2018). Updated Molecular Testing Guideline for the Selection of Lung Cancer Patients for Treatment With Targeted Tyrosine Kinase Inhibitors: Guideline From the College of American Pathologists, the International Association for the Study of Lung Cancer, and the Association for Molecular Pathology. Arch. Pathol. Lab. Med..

[5] Gridelli C., De Marinis F., Di Maio M., Cortinovis D., Cappuzzo F., Mok T. (2011). Gefitinib as first-line treatment for patients with advanced non-small-cell lung cancer with activating epidermal growth factor receptor mutation: Review of the evidence. Lung Cancer.

[6] Kumarakulasinghe N.B., van Zanwijk N., Soo R.A. (2015). Molecular targeted therapy in the treatment of advanced stage non-small cell lung cancer (NSCLC). Respirology.

[7] Shea M., Costa D.B., Rangachari D. (2016). Management of advanced non-small cell lung cancers with known mutations or rearrangements: Latest evidence and treatment approaches. Ther. Adv. Respir. Dis..

[8] Hanahan D., Weinberg R.A. (2011). Hallmarks of cancer: The next generation. Cell.

[9] Proctor M.J., Morrison D.S., Talwar D., Balmer S.M., Fletcher C.D., O’Reilly D.S., Foulis A.K., Horgan P.G., McMillan D.C. (2011). A comparison of inflammation-based prognostic scores in patients with cancer. A Glasgow Inflammation Outcome Study. Eur. J. Cancer.

[10] Ueno H., Hawrylowicz C.M., Banchereau J. (2007). Immunological intervention in human diseases. J. Transl. Med..

[11] Yang R., Chang Q., Meng X., Gao N., Wang W. (2018). Prognostic value of Systemic immune-inflammation index in cancer: A meta-analysis. J. Cancer.

[12] Wang Y., Li Y., Chen P., Xu W., Wu Y., Che G. (2019). Prognostic value of the pretreatment systemic immune-inflammation index (SII) in patients with non-small cell lung cancer: A meta-analysis. Ann. Transl. Med..

[13] Zhang Y., Chen B., Wang L., Wang R., Yang X. (2019). Systemic immune-inflammation index is a promising noninvasive marker to predict survival of lung cancer: A meta-analysis. Medicine.

[14] Cupp M.A., Cariolou M., Tzoulaki I., Aune D., Evangelou E., Berlanga-Taylor A.J. (2020). Neutrophil to lymphocyte ratio and cancer prognosis: An umbrella review of systematic reviews and meta-analyses of observational studies. BMC Med..

[15] Chan S.W.S., Smith E., Aggarwal R., Balaratnam K., Chen R., Hueniken K., Fazelzad R., Weiss J., Jiang S., Shepherd F.A. (2021). Systemic Inflammatory Markers of Survival in Epidermal Growth Factor-Mutated Non-Small-Cell Lung Cancer: Single-Institution Analysis, Systematic Review, and Meta-analysis. Clin. Lung Cancer.

[16] Yun N.K., Rouhani S.J., Bestvina C.M., Ritz E.M., Gilmore B.A., Tarhoni I., Borgia J.A., Batus M., Bonomi P.D., Fidler M.J. (2021). Neutrophil-to-Lymphocyte Ratio Is a Predictive Biomarker in Patients with Epidermal Growth Factor Receptor (*EGFR*) Mutated Advanced Non-Small Cell Lung Cancer (NSCLC) Treated with Tyrosine Kinase Inhibitor (TKI) Therapy. Cancers.

[17] Prelaj A., Rebuzzi S.E., Pizzutilo P., Bilancia M., Montrone M., Pesola F., Longo V., Del Bene G., Lapadula V., Cassano F. (2020). EPSILoN: A Prognostic Score Using Clinical and Blood Biomarkers in Advanced Non-Small-cell Lung Cancer Treated With Immunotherapy. Clin. Lung Cancer.

[18] Cuaron J., Dunphy M., Rimner A. (2012). Role of FDG-PET scans in staging, response assessment, and follow-up care for non-small cell lung cancer. Front. Oncol..

[19] de Geus-Oei L.F., van der Heijden H.F., Corstens F.H., Oyen W.J. (2007). Predictive and prognostic value of FDG-PET in nonsmall-cell lung cancer: A systematic review. Cancer.

[20] Yu M., Chen S., Hong W., Gu Y., Huang B., Lin Y., Zhou Y., Jin H., Deng Y., Tu L. (2019). Prognostic role of glycolysis for cancer outcome: Evidence from 86 studies. J. Cancer Res. Clin. Oncol..

[21] Park S.Y., Cho A., Yu W.S., Lee C.Y., Lee J.G., Kim D.J., Chung K.Y. (2015). Prognostic value of total lesion glycolysis by ^18^F-FDG PET/CT in surgically resected stage IA non-small cell lung cancer. J. Nucl. Med..

[22] McGranahan N., Swanton C. (2017). Clonal Heterogeneity and Tumor Evolution: Past, Present, and the Future. Cell.

[23] Bailly C., Bodet-Milin C., Bourgeois M., Gouard S., Ansquer C., Barbaud M., Sébille J.C., Chérel M., Kraeber-Bodéré F., Carlier T. (2019). Exploring Tumor Heterogeneity Using PET Imaging: The Big Picture. Cancers.

[24] Piñeiro-Fiel M., Moscoso A., Pubul V., Ruibal Á., Silva-Rodríguez J., Aguiar P. (2021). A Systematic Review of PET Textural Analysis and Radiomics in Cancer. Diagnostics.

[25] Bashir U., Azad G., Siddique M.M., Dhillon S., Patel N., Bassett P., Landau D., Goh V., Cook G. (2017). The effects of segmentation algorithms on the measurement of ^18^F-FDG PET texture parameters in non-small cell lung cancer. EJNMMI Res..

[26] Chen Y.H., Wang T.F., Chu S.C., Lin C.B., Wang L.Y., Lue K.H., Liu S.H., Chan S.C. (2020). Incorporating radiomic feature of pretreatment ^18^F-FDG PET improves survival stratification in patients with *EGFR*-mutated lung adenocarcinoma. PLoS ONE.

[27] Cheng N.M., Fang Y.H., Tsan D.L., Hsu C.H., Yen T.C. (2016). Respiration-Averaged CT for Attenuation Correction of PET Images–Impact on PET Texture Features in Non-Small Cell Lung Cancer Patients. PLoS ONE.

[28] Cook G.J., O’Brien M.E., Siddique M., Chicklore S., Loi H.Y., Sharma B., Punwani R., Bassett P., Goh V., Chua S. (2015). Non-Small Cell Lung Cancer Treated with Erlotinib: Heterogeneity of ^18^F-FDG Uptake at PET-Association with Treatment Response and Prognosis. Radiology.

[29] Tixier F., Hatt M., Valla C., Fleury V., Lamour C., Ezzouhri S., Ingrand P., Perdrisot R., Visvikis D., Le Rest C.C. (2014). Visual versus quantitative assessment of intratumor ^18^F-FDG PET uptake heterogeneity: Prognostic value in non-small cell lung cancer. J. Nucl. Med..

[30] Edge S.B., Compton C.C. (2010). The American Joint Committee on Cancer: The 7th edition of the AJCC cancer staging manual and the future of TNM. Ann. Surg. Oncol..

[31] Bauckneht M., Genova C., Rossi G., Rijavec E., Dal Bello M.G., Ferrarazzo G., Tagliamento M., Donegani M.I., Biello F., Chiola S. (2021). The Role of the Immune Metabolic Prognostic Index in Patients with Non-Small Cell Lung Cancer (NSCLC) in Radiological Progression during Treatment with Nivolumab. Cancers.

[32] Boellaard R., Delgado-Bolton R., Oyen W.J., Giammarile F., Tatsch K., Eschner W., Verzijlbergen F.J., Barrington S.F., Pike L.C., Weber W.A. (2015). FDG PET/CT: EANM procedure guidelines for tumour imaging: Version 2.0. Eur. J. Nucl. Med. Mol. Imaging.

[33] Hellwig D., Graeter T.P., Ukena D., Groeschel A., Sybrecht G.W., Schaefers H.J., Kirsch C.M. (2007). ^18^F-FDG PET for mediastinal staging of lung cancer: Which SUV threshold makes sense?. J. Nucl. Med..

[34] Desseroit M.C., Tixier F., Weber W.A., Siegel B.A., Cheze Le Rest C., Visvikis D., Hatt M. (2017). Reliability of PET/CT Shape and Heterogeneity Features in Functional and Morphologic Components of Non-Small Cell Lung Cancer Tumors: A Repeatability Analysis in a Prospective Multicenter Cohort. J. Nucl. Med..

[35] Zwanenburg A., Vallières M., Abdalah M.A., Aerts H., Andrearczyk V., Apte A., Ashrafinia S., Bakas S., Beukinga R.J., Boellaard R. (2020). The Image Biomarker Standardization Initiative: Standardized Quantitative Radiomics for High-Throughput Image-based Phenotyping. Radiology.

[36] Camp R.L., Dolled-Filhart M., Rimm D.L. (2004). X-tile: A new bio-informatics tool for biomarker assessment and outcome-based cut-point optimization. Clin. Cancer Res..

[37] Park S.Y., Park J.E., Kim H., Park S.H. (2021). Review of Statistical Methods for Evaluating the Performance of Survival or Other Time-to-Event Prediction Models (from Conventional to Deep Learning Approaches). Korean J. Radiol..

[38] Mehta H.B., Mehta V., Girman C.J., Adhikari D., Johnson M.L. (2016). Regression coefficient-based scoring system should be used to assign weights to the risk index. J. Clin. Epidemiol..

[39] Hua X., Chen J., Wu Y., Sha J., Han S., Zhu X. (2019). Prognostic role of the advanced lung cancer inflammation index in cancer patients: A meta-analysis. World J. Surg. Oncol..

[40] Shoji F. (2020). Clinical impact of the systemic immune-inflammation index in non-small cell lung cancer patients. Ann. Transl. Med..

[41] Deng C., Zhang N., Wang Y., Jiang S., Lu M., Huang Y., Ma J., Hu C., Hou T. (2019). High systemic immune-inflammation index predicts poor prognosis in advanced lung adenocarcinoma patients treated with *EGFR*-TKIs. Medicine.

[42] Li H., Wang G., Zhang H., Song X., Cao J., Zhang X., Xue R., Wang W., Jia S., Li Z. (2019). Prognostic role of the systemic immune-inflammation index in brain metastases from lung adenocarcinoma with different *EGFR* mutations. Genes Immun..

[43] Shoji F. (2019). Clinical impact of preoperative immunonutritional status in patients undergoing surgical resection of lung cancer. J. Thorac. Dis..

[44] Jiang S., Wang S., Wang Q., Deng C., Feng Y., Ma F., Ma J., Liu X., Hu C., Hou T. (2021). Systemic Inflammation Response Index (SIRI) Independently Predicts Survival in Advanced Lung Adenocarcinoma Patients Treated with First-Generation *EGFR*-TKIs. Cancer Manag. Res..

[45] Ju Q., Huang T., Zhang Y., Wu L., Geng J., Mu X., Yan T., Zhang J. (2021). Systemic immune-inflammation index predicts prognosis in patients with different *EGFR*-mutant lung adenocarcinoma. Medicine.

[46] Yucel S., Bilgin B. (2021). The prognostic values of systemic immune-inflammation index and derived neutrophil-lymphocyte ratio in *EGFR*-mutant advanced non-small cell lung cancer. J. Oncol. Pharm. Pract.

[47] Moon S.H., Kim J., Joung J.G., Cha H., Park W.Y., Ahn J.S., Ahn M.J., Park K., Choi J.Y., Lee K.H. (2019). Correlations between metabolic texture features, genetic heterogeneity, and mutation burden in patients with lung cancer. Eur. J. Nucl. Med. Mol. Imaging.

[48] Wu G., Jochems A., Refaee T., Ibrahim A., Yan C., Sanduleanu S., Woodruff H.C., Lambin P. (2021). Structural and functional radiomics for lung cancer. Eur. J. Nucl. Med. Mol. Imaging.

[49] Traverso A., Wee L., Dekker A., Gillies R. (2018). Repeatability and Reproducibility of Radiomic Features: A Systematic Review. Int. J. Radiat Oncol. Biol. Phys..

[50] Lue K.H., Chu S.C., Wang L.Y., Chen Y.C., Li M.H., Chang B.S., Chan S.C., Chen Y.H., Lin C.B., Liu S.H. (2021). Tumor glycolytic heterogeneity improves detection of regional nodal metastasis in patients with lung adenocarcinoma. Ann. Nucl. Med..

[51] Xu H., Lv W., Zhang H., Ma J., Zhao P., Lu L. (2021). Evaluation and optimization of radiomics features stability to respiratory motion in ^18^F-FDG 3D PET imaging. Med. Phys..

[52] Nordquist L.T., Simon G.R., Cantor A., Alberts W.M., Bepler G. (2004). Improved survival in never-smokers vs. current smokers with primary adenocarcinoma of the lung. Chest.

[53] Toh C.K., Gao F., Lim W.T., Leong S.S., Fong K.W., Yap S.P., Hsu A.A., Eng P., Koong H.N., Thirugnanam A. (2006). Never-smokers with lung cancer: Epidemiologic evidence of a distinct disease entity. J. Clin. Oncol..

[54] Bryant A., Cerfolio R.J. (2007). Differences in epidemiology, histology, and survival between cigarette smokers and never-smokers who develop non-small cell lung cancer. Chest.

[55] Ou S.H., Ziogas A., Zell J.A. (2009). Asian ethnicity is a favorable prognostic factor for overall survival in non-small cell lung cancer (NSCLC) and is independent of smoking status. J. Thorac. Oncol..

[56] Kawaguchi T., Takada M., Kubo A., Matsumura A., Fukai S., Tamura A., Saito R., Maruyama Y., Kawahara M., Ignatius Ou S.H. (2010). Performance status and smoking status are independent favorable prognostic factors for survival in non-small cell lung cancer: A comprehensive analysis of 26,957 patients with NSCLC. J. Thorac. Oncol..

[57] Gettinger S., Hellmann M.D., Chow L.Q.M., Borghaei H., Antonia S., Brahmer J.R., Goldman J.W., Gerber D.E., Juergens R.A., Shepherd F.A. (2018). Nivolumab Plus Erlotinib in Patients With *EGFR*-Mutant Advanced NSCLC. J. Thorac. Oncol..

[58] Polley M.C., Dignam J.J. (2021). Statistical Considerations in the Evaluation of Continuous Biomarkers. J. Nucl. Med..

[59] Wu S.G., Shih J.Y. (2018). Management of acquired resistance to *EGFR* TKI-targeted therapy in advanced non-small cell lung cancer. Mol. Cancer.

[60] Du X., Yang B., An Q., Assaraf Y.G., Cao X., Xia J. (2021). Acquired resistance to third-generation *EGFR*-TKIs and emerging next-generation *EGFR* inhibitors. Innovation.

[61] Gieszer B., Megyesfalvi Z., Dulai V., Papay J., Kovalszky I., Timar J., Fillinger J., Harko T., Pipek O., Teglasi V. (2021). *EGFR* variant allele frequency predicts *EGFR*-TKI efficacy in lung adenocarcinoma: A multicenter study. Transl. Lung Cancer Res..

[62] Zhang Y., Sheng J., Kang S., Fang W., Yan Y., Hu Z., Hong S., Wu X., Qin T., Liang W. (2014). Patients with exon 19 deletion were associated with longer progression-free survival compared to those with L858R mutation after first-line *EGFR*-TKIs for advanced non-small cell lung cancer: A meta-analysis. PLoS ONE.

[63] Sheng M., Wang F., Zhao Y., Li S., Wang X., Shou T., Luo Y., Tang W. (2016). Comparison of clinical outcomes of patients with non-small-cell lung cancer harbouring epidermal growth factor receptor exon 19 or exon 21 mutations after tyrosine kinase inhibitors treatment: A meta-analysis. Eur. J. Clin. Pharmacol..

[64] Fournier L., Costaridou L., Bidaut L., Michoux N., Lecouvet F.E., de Geus-Oei L.F., Boellaard R., Oprea-Lager D.E., Obuchowski N.A., Caroli A. (2021). Incorporating radiomics into clinical trials: Expert consensus endorsed by the European Society of Radiology on considerations for data-driven compared to biologically driven quantitative biomarkers. Eur. Radiol..

